# A thermal gradient modulates the oxidative metabolism and growth of human keratinocytes

**DOI:** 10.1002/2211-5463.12303

**Published:** 2017-10-24

**Authors:** Marta Viano, Daniela Alotto, Alessia Aillon, Carlotta Castagnoli, Francesca Silvagno

**Affiliations:** ^1^ Dipartimento di Oncologia University of Torino Italy; ^2^ Dipartimento di Chirurgia Generale e Specialistiche Banca della Cute University of Torino Italy

**Keywords:** ERβ, keratinocyte, low temperature, mitochondrial ATP, proliferation

## Abstract

During their spatial and differentiative progression, keratinocytes face a thermal gradient, from 37 °C in the proliferating basal layer to 32 °C found in skin surface. In our study, we hypothesized that this difference in temperature must be balanced by increasing the heat produced during respiratory activity. We demonstrated that at 33 °C human primary keratinocytes and HaCaT cells raised mitochondrial energy metabolism, but not glycolytic activity. At 33 °C, the increased mitochondrial ATP synthesis was associated with a strong induction of the modulator of the respiratory chain estrogen receptor β, whereas uncoupling protein 1 expression was not changed. The enhanced mitochondrial oxidative metabolism was accompanied by a remarkable reduction in proliferation. These results suggest that environmental temperature can modulate the energy metabolism and proliferation of human keratinocytes.

**Abbreviations**
ERβ: estrogen receptor β
LDH: lactate dehydrogenase
TCA cycle: tricarboxylic acid cycle
TRPM8: transient receptor potential cation channel subfamily M member 8
UCP1: uncoupling protein 1

Epidermis is the most external compartment of skin, and it is formed by several layers of keratinocytes, exhibiting different metabolic and structural phenotypes. This stratification ranges from the basal proliferating compartment up to the spinous and the granular layers, where differentiated keratinocytes produce lipids and proteins that will form the protective epidermal barrier. The outer lucidum and cornified layers are mainly composed by remnants of dead cells that have secreted lipids and keratins. Along their migration toward the upper layers, keratinocytes adapt their metabolism in order to sustain the specialized functions required by each layer. Several pathways involved in the fine‐tuning of the balance between proliferation, differentiation, and death of keratinocytes have been described 1, 2, 3, 4. The main mechanisms appear to be mediated by nuclear transcriptional modulation and calcium signaling. A further interesting feature of human skin is that keratinocytes are exposed to a thermal gradient throughout epidermal layers, which ranges from 37 °C of vascularized derma to a superficial epidermal temperature that has been calculated around 32 °C in comfortable conditions 5. Endotherms respond to temperature variations in the environment by adjusting metabolic heat production and maintain a constant body temperature of 37 °C by adaptive thermogenesis. Intracellular heat production is energetically demanding but is necessary in order to maintain the 37 °C at which the biochemical reactions proceed at a speed compatible with life. In fact at the human body temperature, the enzyme's catalytic activity is at its greatest in human cells. While the central control of thermogenesis has been investigated 6, the effects of thermal variation on the metabolism of single tissues are not well described, except for the thermogenic brown adipose tissue where the process of mitochondrial heat production is mediated by the uncoupling of respiratory chain from ATP synthesis 7, 8. The influence of the temperature on skin has been evaluated with respect to differentiation: The keratinocyte differentiation is a cold‐modulated process, as the transient mild cold exposure optimizes the differentiation of cultured keratinocytes 9; however, the effects of temperature variation on keratinocyte metabolism have not been investigated yet.

In this work, we assume that the thermal gradient encountered by keratinocytes in epidermis can induce the heat production in the powerhouse of the cell that is the mitochondrial compartment. First, we put forward a theoretical evaluation of the energy necessary to contrast the thermal dissipation and we evaluate the increase in oxidative catabolism required for this purpose. Our biochemical considerations are then supported by the experimental evidences obtained in a model that mimics the thermal gradient encountered by epidermal cell layers. In our experimental setup, the human primary keratinocytes and the human cell line HaCaT were kept either at 37 °C or at 33 °C and their energy metabolism and proliferation were investigated.

## Materials and methods

### Cell culture and treatment

Human primary keratinocytes were obtained from Banca della Cute, AOU Città della Salute e della Scienza, Torino, Italy, and were prepared as previously reported 10. A written informed consent was obtained from all donors of skin grafts. Briefly, the cells were grown on a irradiated 3T3‐J2 layer in Dulbecco's modified Eagle medium (DMEM; Gibco, Paisley, UK) and 10% FBS (New Zealand origin) supplemented with 5 μg·mL^−1^ insulin (Sigma‐Aldrich, St. Louis, MO, USA), 0.4 μg·mL^−1^ hydrocortisone (Sigma‐Aldrich), 10^*−*10^ m cholera toxin (Sigma‐Aldrich), 2 *×* 10^*−*10^ m triiodothyronine (Sigma‐Aldrich), 5 μg·mL^−1^ apo‐transferrin (Invitrogen Life Technologies, Carlsbad, CA, USA), 2 mm glutamine (Sigma‐Aldrich), and 50 UI·mL^−1^ penicillin/streptomycin antibiotic solution. The flasks were incubated at 37 °C and 33 °C in 5% CO_2_. At the first medium change (5 days after the initial plating), the medium was further supplemented with 10 ng·mL^−1^ epidermal growth factor (Sigma‐Aldrich) and changed every 48*−*72 h.

The immortalized human epidermal keratinocyte cell line HaCaT was purchased from American Type Culture Collection (ATCC, Manassas, VA, USA), and the cells were cultured in DMEM that had been supplemented with 10% FBS and 1% antibiotics (penicillin/streptomycin; Sigma‐Aldrich) at 37 and 33 °C in a humidified atmosphere containing 5% CO_2_.

The same number of cells was seeded for each experimental condition, and after 10 days, all the cells were counted or harvested for biochemical analysis. When counted, the cells were trypsinized and collected by centrifugation. An aliquot of collected cells was combined with trypan blue dye at a concentration of 0.04% (w/v) and analyzed microscopically on a Burker counting chamber. Blue nonviable cells, very limited in number, were excluded from the count.

### Extract preparation and western blotting analyses

Subcellular fractionation and western blotting analyses were carried out as previously described 11. Lysates were subjected to differential centrifugation to isolate the mitochondrial fraction. Proteins were extracted from mitochondria by incubation in boiling sample buffer followed by sonication. Thirty microgram of each fraction was separated using 10% SDS/PAGE and analyzed by western blotting. The proteins were immunostained with the indicated primary antibodies for 1 h at room temperature, and detection of the proteins of interest was performed using peroxidase‐conjugated secondary antibodies (Pierce, Rockford, IL, USA), followed by ECL detection (ECL detection kit; Perkin Elmer Life Science, Foster City, CA USA). The mouse antibody anti‐estrogen receptor β (anti‐ERβ) (sc‐1531) was from Santa Cruz (Santa Cruz, CA, USA; the rabbit anti‐uncoupling protein 1 (anti‐UCP1; U6382) was from Sigma‐Aldrich. The anti‐VDAC (anti‐porin 31HL) monoclonal antibody was purchased from Calbiochem (La Jolla, CA, USA).

### Evaluation of mitochondrial ATP levels

After ten days of incubation at 37 or 33 °C, the amount of ATP in mitochondria, prepared by subcellular fractionation, was measured with the ATP Bioluminescent Assay Kit (FL‐AA; Sigma), using a Synergy HT Multi‐Mode Microplate Reader (BioTek Instruments Inc., Winooski, VT, USA). ATP was quantified as relative light units (RLU); data were converted into nmol ATP per mg mitochondrial proteins and were also expressed as the percentage of their respective counterpart kept at 37 °C (relative ATP).

### Measurement of intracellular lactate dehydrogenase (LDH) activity

After ten days of incubation at 37 or 33 °C, the cells were harvested by scraping and sonicated on ice with two 10‐s bursts. Aliquots of cell lysate were supplemented with a reaction mixture for the measurement of LDH, as previously described 12. Enzymatic activity, measured spectrophotometrically as absorbance variation at 340 nm (37 °C), was expressed as μmol NADH oxidized per min per mg cell protein.

### Bands quantification and statistical analysis

Bands from protein electrophoresis were quantified by scanning digital densitometry using an imagej software analysis (ImageJ version 1.29; Sun Microsystems Inc., Palo Alto, CA, USA).

The data are presented as the means ± SD. Statistical analysis of the data was performed using an unpaired, two‐tailed Student's *t*‐test. *P* < 0.05 was considered to be significant.

## Results

### Biochemical analysis of the cellular response to thermal variation

When a cell is exposed to temperatures cooler than the standard 37 °C, cellular heat loss is compensated by heat production, with the aim of restoring 37 °C. In other words, the cell opposes to the temperature drop by increasing heat production. Knowing the thermal variation in the environment, we can calculate the energy absorbed (enthalpy) by the cell to maintain 37 °C; the following formula can be used:

Q=c·m·ΔT

where *Q* is the enthalpy measured in kilocalories (kcal); *c* is the specific heat capacity measured in kcal·kg^−1^·K^−1^; *m* is the mass of the cell that the heat energy is transferred into, measured in kg; and Δ*T* is the difference in temperature between environmental temperature and the intracellular endpoint temperature of 37 °C, measured in kelvin (K).

Q can be calculated for a cell incubated at 33 °C (*Q*_33_) and at 36.6 °C (*Q*_36,6_). 33 °C is the temperature found in the outer layer of living keratinocytes, whereas 36.6 °C is the temperature of the basal layer close to vascularized derma. Considering the cell, a thermal dissipating system there must be a temperature difference of at least 0.4 °C between environment and cell 13, 14 necessary for survival; if we assume that the optimal temperature maintained by the cell is 37 °C, then we must define the temperature of a standard incubator as 36.6 °C, a value that is well in the range of precision of the instrument. Therefore in this section, we consider the temperature of standard growth condition as 36.6 °C, whereas in the rest of the article, we refer to this temperature as 37 °C. We consider the thermal difference between the internal 37 °C and the external temperature of a cell living at 33 or 36.6 °C, and we calculate the respective enthalpies:

$$Q_{36.6}=c\cdot m\cdot 0.4\;K$$

$$Q_{33}=c\cdot m\cdot 4\;K$$

The values of *c* and *m* are quite difficult to measure, because of the complexity and lack of homogeneity of the cell composition; however, it is of note that whatever approximate value we choose, it will be the same in both formulas. We decided to consider c as the value of water; water has a high heat capacity (0.999 kcal·kg^−1^·K^−1^ in the range 33–37 °C) compared to other substances because of its hydrogen bonds; therefore, the real value of *c* for a cell will be smaller, due to the weaker Van der Waals interactions keeping the cellular membranes together. As for *m*, we considered a keratinocyte mean cellular volume of 1 × 10^4^ μm^3^ (as calculated in 15), which considering again a water solution gives a value of 1 × 10^−5^ kg.

The previous equations become:

$$Q_{36.6}=0.999\;\text{kcal}\cdot {\text{kg}}^{-1}\cdot \;K^{-1}\cdot \left(1\cdot 10^{-5}\text{kg}\right)\cdot 0.4\;K=4\cdot 10^{-6}\text{kcal}$$

$$Q_{33}=0.999\;\text{kcal}\cdot {\text{kg}}^{-1}\cdot \;K^{-1}\cdot \left(1\cdot 10^{-5}\text{kg}\right)\cdot 4\;K=4\cdot 10^{-5}\text{kcal}$$

The calories absorbed by the cell to increase the internal temperature are mainly generated in the mitochondrial compartment, where reducing units are oxidized by respiratory chain. Here, chemical energy is partly converted in ATP biosynthesis, partly dissipated as heat. Many studies have demonstrated that the oxidation of nutrients at the level of mitochondrial respiratory activity is the primary heat‐releasing process (reviewed in Ref. 16). However, high ratios of glycolysis to oxidative metabolism can result in a significant heat production from glycolysis in isolated cells, partly due to the net pH change 17.

Considering the mitochondrial oxidative metabolism as the main aerobic energy metabolism, the *in vivo* enthalpy of NADH oxidation has been calculated as −61.6 kcal·mol^−1^, an energy that is transformed by the cell to 2.5 mol of ATP and 49.85 kcal·mol^−1^ lost as heat dissipation 18. As consequence, one can calculate how many moles of NADH (N) are oxidized to produce Q:

$$N_{36.6}=4\cdot 10^{-6}\;\text{kcal}/49.85\;\text{kcal}\cdot {\text{mol}}^{-1}=0.8\cdot 10^{-7}\text{mol}$$

$$N_{33}=4\cdot 10^{-5}\;\text{kcal}/49.85\;\text{kcal}\cdot {\text{mol}}^{-1}=0.8\cdot 10^{-6}\text{mol}$$

This calculation is very theoretical, because it is based on the assumption that oxidative phosphorylation and substrate‐level phosphorylation are tightly coupled to the oxidation of the substrate. In real tissues, this is barely the case; in fact, it has been estimated that proton leak accounts for 10–30% of oxygen consumption in several tissues such as skeletal muscle 19, liver 20, and heart 21. Therefore, the calculated N moles required to maintain the 37 °C would be even lower. In our biochemical consideration, however, the main conclusion we put forward is that when the environmental temperature drops to 33°, as it is the case of the most external epidermal layer, the cells adjust their oxidative metabolism and consume up to ten times more substrates to balance heat dispersion than the cells living at 37 °C.

Recent papers have shown that the cells are in balance between a biosynthetic proliferative status and a catabolic quiescent phenotype (reviewed in Ref. 22). When a cell is forced by the environment to enhance oxidative catabolism, this will affect the availability of intermediates for biosynthesis and will reduce growth. Based on our biochemical analysis, we postulate that the decrease in external temperature will induce the mitochondrial respiration and the coupled oxidative phosphorylation for thermoregulatory purpose, and to support this effort, the cell will enhance oxidative catabolism and will reduce proliferation.

Therefore, we set forth to measure the energy metabolism and growth of our cellular models, the human primary keratinocytes, and the human immortalized cell line HaCaT kept either at 37 °C or at 33 °C.

### The mitochondrial metabolism is increased at 33 °C, whereas the glycolytic flux is not affected

**Figure 1:**
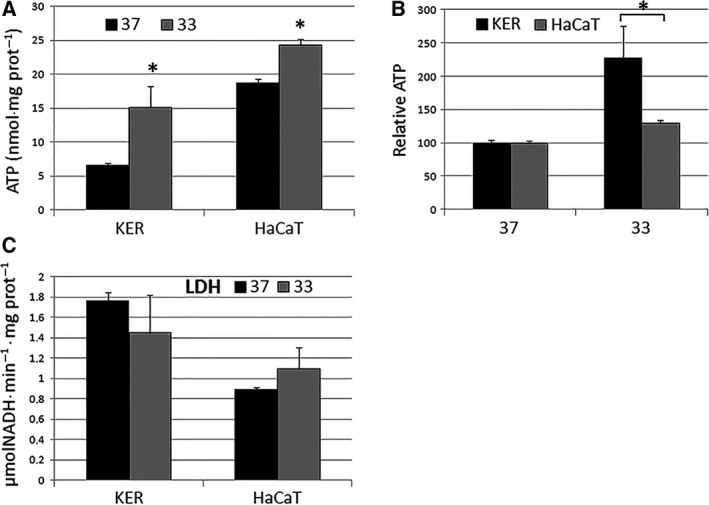
The effect of the temperature on cellular energy metabolism. Primary keratinocytes (KER) and HaCaT cells were incubated for 10 days at 37 and 33 °C, and the levels of mitochondrial ATP were measured by a chemiluminescence‐based assay. (A) Data were expressed as nmol/mg of proteins and (B) as the percentage of their respective counterpart kept at 37 °C (relative ATP), in order to emphasize the higher induction of ATP synthesis in KER compared to HaCaT. (C) The intracellular LDH activity was assayed in the same conditions. Measurements were taken in triplicate, and data are presented as means ± SD (*n* = 3). *P < 0.05 compared to the cells at 37 °C.

To ascertain whether a change in external temperature would cause an alteration in energy metabolism, we tested the mitochondrial oxidative activity as mitochondrial ATP production, whereas the cytoplasmic glycolytic flux was evaluated indirectly as the activity of the downstream enzyme lactate dehydrogenase (LDH) in cell lysates. The basis for this approach is that it has been argued, at least for some cell types 23, 24, that any increase in the rate of glycolysis is accompanied by an increase in the amount of LDH enzyme. As there is evidence that the lactate/pyruvate reaction is always at equilibrium 25 even under aerobic conditions, the amount of LDH activity is assumed here to give a measure of the glycolytic rate under any conditions. Both primary cultures of human keratinocytes and highly proliferating human keratinocytes HaCaT were exposed for 10 days to 33 and 37 °C. The results of the metabolic analysis are shown in Fig. 1. We found an increased production of mitochondrial ATP when all these cells were kept at 33 °C (Fig. 1A), and the effect was more pronounced in primary keratinocytes, because the increase in ATP levels is significantly higher in keratinocytes compared to HaCaT (Fig. 1B). By contrast, LDH activity did not change whether the cells were maintained at 33 or 37 °C (Fig. 1C). We hypothesized that the measured increase in mitochondrial ATP levels was not due to a reduced utilization of cellular ATP. The regulatory mechanisms of mitochondrial respiration are very complex and are influenced by many factors; theories of the mechanism of mitochondrial respiratory control envisage that state 3 is controlled by the ATP turnover and by the substrate oxidation (including substrate uptake, modulation of electron transport chain complexes, ubiquinol/ubiquinone ratios, and oxygen levels) 26, 27. When ATP is not consumed, the ATP/ADP ratio rises and respiration slows to state 4. Moreover, the studies by Kadenbach and colleagues have proposed that ATP exerts a negative allosteric modulation of the activity of complex IV 28, 29. For all these reasons, a lower expenditure of ATP should not increase the amount of mitochondrial ATP.

Our results showed that the drop in environmental temperature enhanced the mitochondrial levels of ATP, possibly as a result of an increased respiratory chain activity, whereas the cytoplasmatic production of ATP was not involved in the thermoregulatory mechanism.

### In primary keratinocytes, the exposure to 33 °C induces the expression of the estrogen receptor β, a known modulator of the electron transport chain

**Figure 2:**
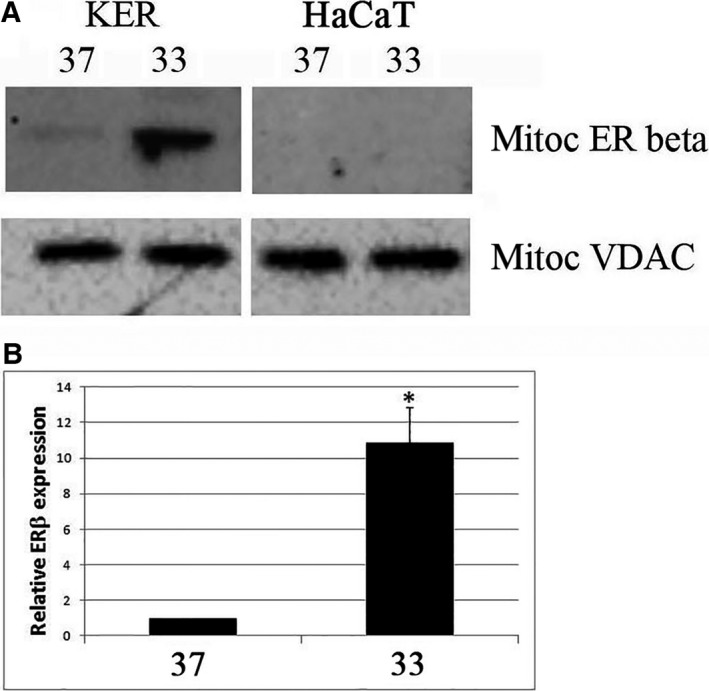
The influence of the temperature on the expression of a modulator of the mitochondrial respiratory chain. Primary keratinocytes (KER) and HaCaT cells were incubated for 10 days at 37 and 33 °C, and the levels of mitochondrial estrogen receptor beta (ERβ) were analyzed by western blotting in mitochondrial extracts. VDAC levels were used as internal controls for protein loading. (A) The blots are representative of a set of three independent experiments. (B) Bands from KER extracts were quantified, normalized for loading as a ratio to VDAC expression, and data plotted on graph as values relative to control. Data represent the mean ± SD of three independent experiments. *P < 0.05 compared to the cells at 37 °C.

Because the observed increase in mitochondrial ATP could be the sign of an increased respiratory activity, we sought to find a mechanism of control able to modulate the respiratory chain. We focused on the transcriptional control exerted by many steroid hormones on mitochondrial gene sets; in particular, we investigated the estrogen receptor beta, because this receptor is a known positive modulator of the respiratory chain and its expression has been described in human keratinocytes and skin 30, 31, 32, 33. In regular cultural conditions at 37 °C, the protein was barely detectable, but very interestingly we found the increased expression of estrogen receptor beta in the mitochondrial extracts of primary keratinocytes exposed to 33 °C (Fig. 2). The same induction was not observed in HaCaT cells, which seem to have lost the possibility of inducing the receptor and therefore must modulate the respiratory chain with different mechanisms. This difference is not surprising, as it is known that tumor cells or immortalized cell lines do not express ERβ and the possibility of modulating this receptor has never been demonstrated in cancer cells.

### Uncoupling protein UCP1 is not modulated by mild temperature in primary keratinocytes

**Figure 3:**
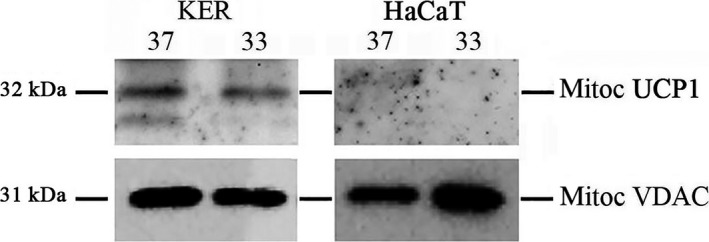
The impact of the temperature on a key protein of the uncoupling process. After 10 days at 37 and 33 °C, the mitochondrial extracts from primary keratinocytes (KER) and HaCaT cells were analyzed by western blotting to detect the expression of the mitochondrial uncoupling protein 1 (UCP1). VDAC levels were used as internal controls for protein loading. The reported molecular weights of the two proteins are indicated. The blots are representative of a set of three independent experiments.

In addition to the increased respiratory activity detected as mitochondrial ATP synthesis, another source of heat production could be the process of uncoupling. The thermal control is mediated by the uncoupling proteins (UCP) in brown adipose tissue, which is specialized in thermogenesis and uses the modulation of UCPs as the main mechanism to perform its task. Also in keratinocytes, the expression of UCPs has been described 34, although their role in keratinocyte metabolism has not been characterized. In particular, UCP1 has been detected by immunohistochemistry in various sites of human epidermis and in cultured human keratinocytes 34. By western blotting assay, we were able to detect the presence of UCP1 only in mitochondrial extracts of primary keratinocytes, but not in HaCaT cells. In any case, UCP1 expression was not altered when the cells were exposed to the coldest temperature (Fig. 3). These observations suggest that UCP1 is not a player of thermoregulation in our model of thermal dissipation, although we cannot rule out the involvement of additional uncoupling mechanisms.

### The proliferation of both primary keratinocytes and HaCaT cells is hampered by low temperature

**Figure 4:**
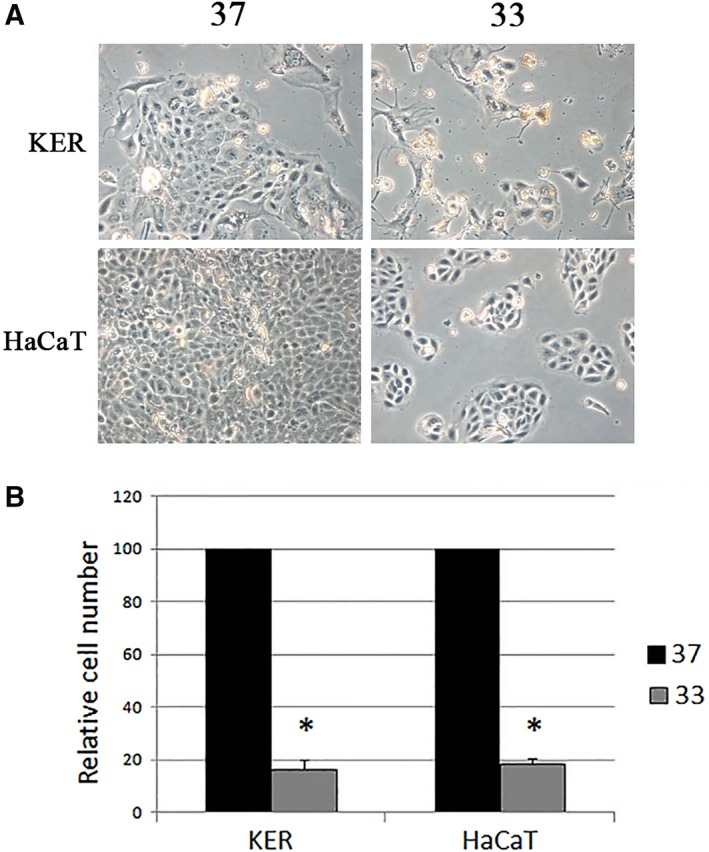
The effect of the temperature on cell morphology and growth. Primary keratinocytes (KER) and HaCaT cells were grown for 10 days at 37 and 33 °C. (A) Representative pictures of the cells at different temperatures. (B) The cells in each dish were counted and the values of the cells kept at 33 °C are expressed as the percentage of their counterpart grown at 37 °C. The data are expressed as the means ± SD of three independent experiments. *P < 0.05 compared to the cells at 37 °C.

After ten days of exposure to different temperatures, we evaluated the proliferation of both primary keratinocytes and HaCaT by counting the number of cells in each dish. No loss of cell viability was detected using trypan blue exclusion assay, and the morphology of the cells resulted unchanged by the different temperatures (Fig. 4A). At 33 °C, all cells decreased dramatically their proliferation and at the end of the treatment were quantified as less than 20% of their counterpart kept at 37 °C. Figure 4B shows the results of the quantification carried out for both cell types.

### A model of the biochemical analysis of thermogenesis in keratinocytes

**Figure 5:**
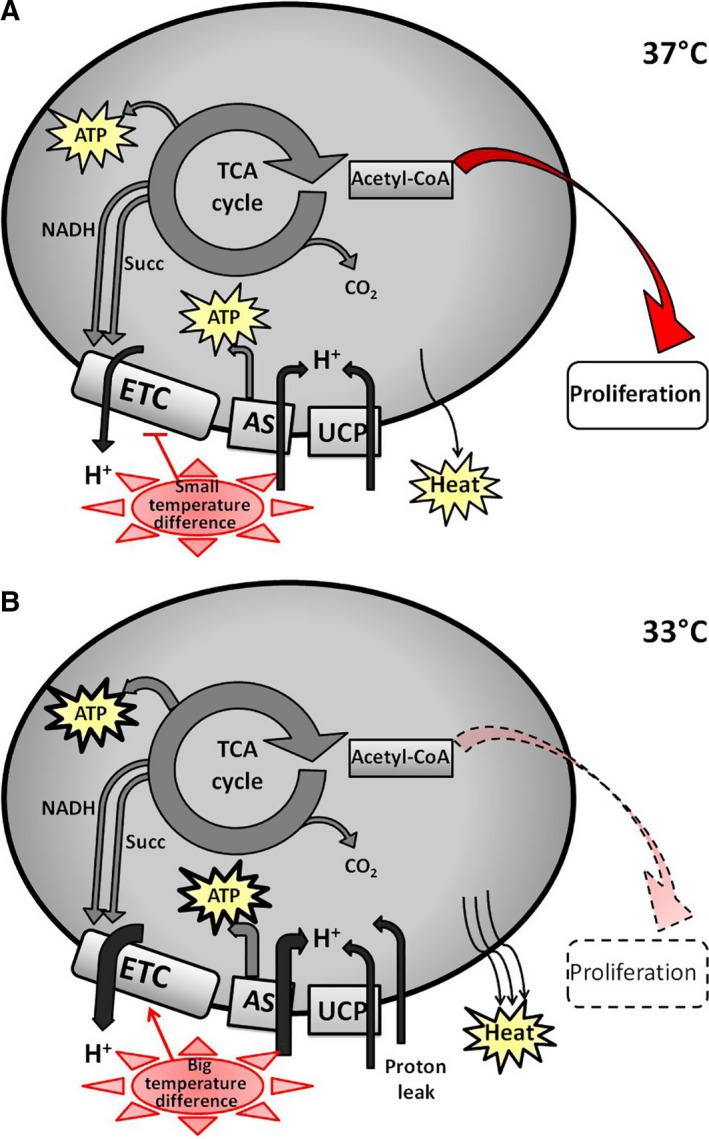
A working model of the proposed molecular mechanisms underlying the effect of external temperature on metabolism of human primary keratinocytes and HaCaT cells. (A) At 37 °C, the mitochondrial metabolism supports both the synthesis of ATP and the rerouting of intermediates (acetyl‐CoA) to support proliferation. (B) At 33 °C, the thermal gradient between internal and external temperature is balanced by heat production at the electron transport chain (ETC) site, either by uncoupling or by proton leak; the acetyl‐CoA is consumed in the tricarboxylic acid cycle (TCA cycle), the oxidative metabolism prevails over the biosynthetic process, and the cell reduces proliferation. AS, ATP synthase; UCP, uncoupling protein; Succ, succinate.

The striking difference in cell number together with the net increase in mitochondrial ATP levels and the induction of the respiratory chain modulator ERβ were observations supporting our initial theoretical considerations on the metabolic effects of a thermal gradient on keratinocytes. At 33 °C, we found a remarkable increase in mitochondrial ATP, which is produced by oxidative phosphorylation. On the other hand, the observed strong induction of mitochondrial ERβ is suggestive of a consequent increase in the respiratory chain, thus coupled to ATP synthesis. The respiratory chain is fed either by cytoplasmic reducing units, and we have indirect evidences that the glycolytic flux is not significantly modulated, or by the reducing units produced by tricarboxylic acid cycle, which moreover provides ATP. In agreement with our theoretical calculation, at 33 °C our experimental results suggested the necessity of inducing mitochondrial respiration to maintain a constant internal temperature, and this could lead the keratinocyte to consume intermediates such as acetyl‐CoA at the expenses of the biosynthetic processes fundamental for proliferation. To illustrate this conclusion, we propose a model that is depicted in Fig. 5. At 37 °C, the difference between internal and external temperature is small and the cell can spare intermediates for biosynthetic purposes sustaining proliferation (Fig. 5A). When the environmental temperature drops to 33 °C, the big temperature difference induces the respiratory activity and the cell dissipates heat to keep the internal 37 °C. Under this thermal stress, the oxidative catabolism is boosted and the availability of intermediates that can be diverted to favor proliferation is reduced (Fig. 5B).

## Discussion

The epidermis undergoes homeostatic regulation as basal proliferating cells execute their program of terminal differentiation and move toward the external surface of skin. Several factors drive this spatial and phenotypic transition; one of the cues encountered across the layers is the cooling of extracellular temperature, which ranges from the 37 °C of vascularized derma to the 32 °C of the outer lipid barrier. In primary keratinocytes, it has been reported that the low temperature slows down cell growth by unclear mechanisms and promotes keratinocyte differentiation; in fact, mild temperature does not enhance the expression of the cell cycle inhibitors, but in the presence of calcium and serum, it induces the expression of differentiation markers 9. To date, no one has investigated how low temperature influences proliferation and differentiation, and most importantly the physiological significance of the external temperature gradient in the differentiation process of keratinocytes. In this study for the first time, we consider the metabolic stress imposed by temperature drop. On theoretical basis, we believe that a difference of only three degrees would evoke a strong request of energy to keep the internal temperature constant; at 33 °C, the cell would oxidize ten times more substrates than a cell kept at 37 °C in the mitochondrial respiratory chain, which is the main producer of cellular energy dissipation as heat. It can be expected that the increased activity of the electron transport chain will raise the mitochondrial production of ATP; indeed, the augmented production of mitochondrial ATP was the first experimental confirmation of our working hypothesis. On the contrary, the activity of the cytosolic enzyme LDH did not change. We considered LDH as a key enzyme in our analysis because its increased activity can be interpreted both as a marker of enhanced glycolytic flux 23, 24 and as a regulator of bioenergetic reserve 25, due to its role in sustaining oxidative phosphorylation through the conversion of lactate into pyruvate. The unaltered activity of LDH suggested that the decrease in temperature did not affect the cytoplasmic energy metabolism. In support of the respiratory chain being the metabolic target of temperature variation, we found that at 33 °C ERβ was upregulated in primary keratinocytes. This receptor is a known positive regulator of the respiratory chain 35, 36, and it is responsible of the antiproliferative and differentiating effects of estrogens on epithelial cells 37, 38, 39, 40, 41, 42. The expression of ERβ is decreased in breast cancer cells compared to the high levels of the receptor found in healthy breast tissue 40, 43, 44, and its downregulation is common in tumors and correlated with malignancy 43, 44, 45, 46, 47. The mechanisms by which the tumor cell can silence ERβ are not known, and there is no treatment effective in restoring the expression and activity of the receptor. This is the first report of a stimulation able to modulate the receptor in proliferating cells and the first study indicating that ERβ is sensitive to temperature variation; the investigation of the molecular differences between primary keratinocytes and HaCaT cells, which are not able to modulate ERβ, could help to understand and possibly overcome the defective ERβ expression in cancer cells.

At 33 °C, the mitochondrial ATP levels increase in HaCaT even in the absence of ERβ modulation, but significantly less than in primary keratinocytes. Because the biological effect of the low temperature is the same (the deceleration of growth is similar for the two cell types), we can hypothesize that in HaCaT cells the uncoupling process might play an important role, although with mechanisms that do not involve the modulation of UCP1. In fact, a significant proportion of protons transferred by the proton pumps of the respiratory chain go back across the inner mitochondrial membrane without being coupled to ATP synthesis (a phenomenon known as proton leak). For example, free fatty acids are natural uncouplers of oxidative phosphorylation acting with a cycling mechanism 48, 49, and also the lipid composition of phospholipid bilayers affects the proton leak 50. Also, the complex IV of the respiratory chain is involved in the regulation of the rate and efficiency of oxidative phosphorylation through intrinsic uncoupling 51. A basal proton leak is always present in cellular systems, accounting for 20–30% of energy loss 50, and it has been suggested that it has a general thermogenic function 52. In our model, the uncoupling explains why the measured increase in mitochondrial ATP is smaller than the calculated theoretical implement of respiratory activity at 33 °C.

In sensory neurons, the mild cold temperature is perceived by thermoreceptors ion channels TRPM8 53, 54, but the role of cold‐sensitive TRP channels is not known in keratinocytes. Recently, the epidermal isoform of TRPM8 (eTRPM8) has been characterized in the membrane of the endoplasmic reticulum; its activity sustains mitochondrial calcium uptake, modulates ATP and superoxide synthesis and it has been concluded that through this receptor the cold temperature controls the balance between proliferation and differentiation of keratinocytes 55. Interestingly, in basal keratinocytes, the level of expression of eTRPM8 is lower than in differentiated keratinocytes that *in vivo* are exposed to 33 °C 55. In their work, Bideaux and colleagues assessed the temperature‐dependent extracellular ATP content and obtained contrasting results depending on the cellular model analyzed (primary keratinocytes, hNEK, or HaCaT cells). In our work, we tested for the first time the modulation of mitochondrial production of ATP triggered by cold temperature and we linked mitochondrial ATP levels with metabolism and proliferation.

In conclusion, we demonstrated that cold temperature influenced the metabolism of keratinocytes by enhancing the oxidative mitochondrial metabolism and not the cytoplasmic glycolysis. In fact, mitochondrial ATP synthesis was induced as the result of the oxidative phosphorylation coupled to the respiratory chain activity controlled by the increased ERβ. The increment of mitochondrial activity was accompanied by sharp impairment of cell growth, which could be due to a metabolic shift from biosynthetic proliferative status toward a catabolic quiescent phenotype.

The consequences of these findings are important to understand the impact of the temperature variations experienced by keratinocytes *in vivo*. Along their migration toward the external surface of epidermis, the keratinocytes sense the temperature gradient generated across the skin layers through the activity of thermoreceptors, and indeed, their expression is augmented in the coldest outer layers of epidermis 55; possibly through a signaling involving mitochondrial calcium 55, the keratinocytes respond to the cooling temperature by increasing the respiratory activity, the production of ATP, and probably the uncoupling process. On the one side, this metabolic activation has a thermogenic purpose, and the cells keep constant the internal temperature; on the other hand, the increased production of ATP benefits the differentiating keratinocytes, which synthesize and actively secrete proteins and lipids of the epidermal barrier. A third outcome of the response to cold is that the metabolic switch toward oxidative catabolism triggered by cold temperature hampers cell growth, in synergy with the ongoing keratinocyte differentiation. Indeed, a recent work has demonstrated that mitochondrial uncoupling drives keratinocyte/epidermal differentiation both *in vitro* and *in vivo* 56.

## Author contributions

FS and CC designed the study. MV, DA, AA, and FS performed the experiments. All authors analyzed the data. FS and CC wrote the manuscript.
